# Supplementary material for: Porcine intraepithelial lymphocytes undergo migration and produce an antiviral response following intestinal virus infection
Source: Commun Biol. 2022 Mar 22;5:252. doi: 10.1038/s42003-022-03205-2 (PMC8941121; doi:10.1038/s42003-022-03205-2)
Supplement: Supplementary file 2 — Supplementary information [file 42003_2022_3205_MOESM2_ESM.pdf]

Supplementary information

Porcine intraepithelial lymphocytes undergo migration and produce an antiviral response following intestinal virus infection

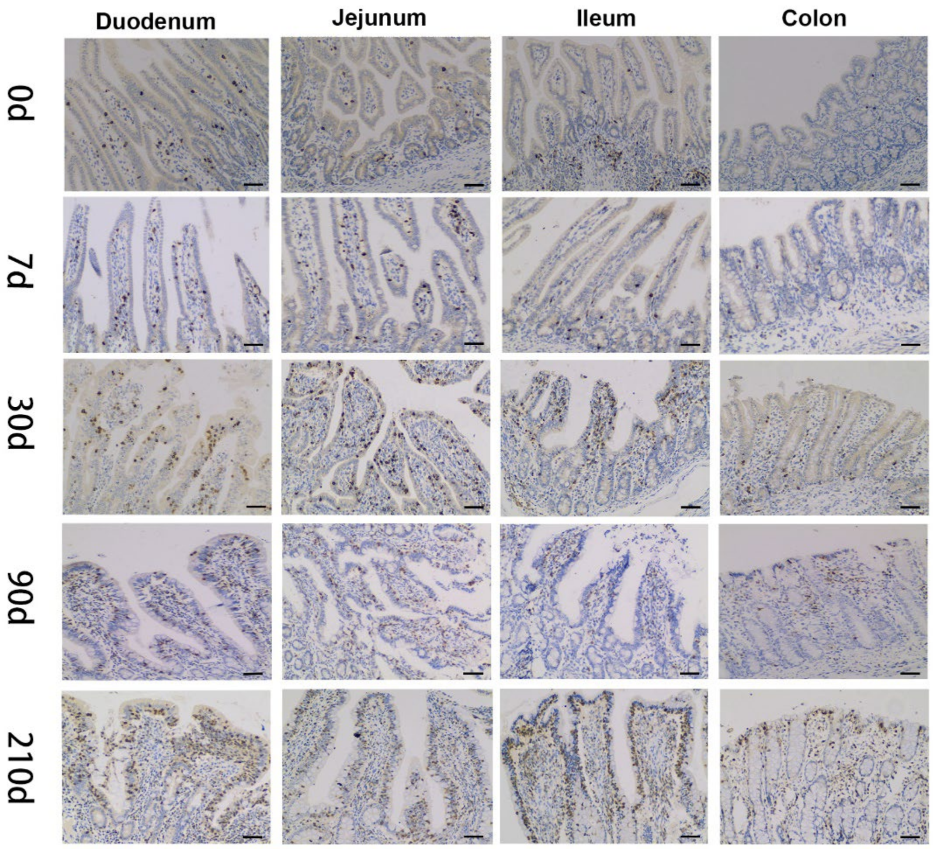

Supplementary Figure 1. The location of CD3⁺ T cells within intestinal epitheliums of pigs in different growth stages. T cell-specific CD3 protein was stained brown. Scale bars, 20 μm.

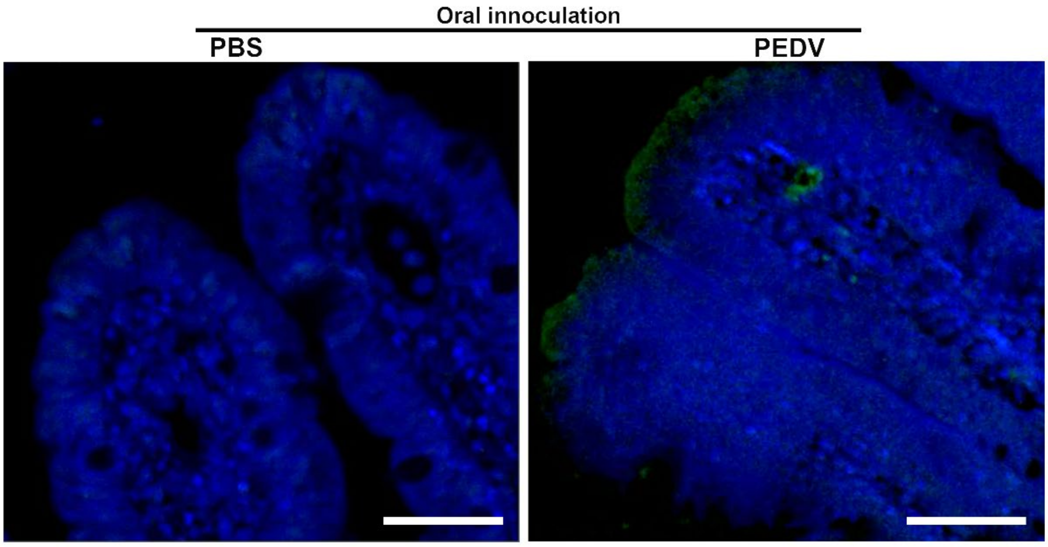

Supplementary Figure 2. Immunofluorescence staining of jejunum from piglets orally inoculated with PEDV. PEDV were immunolabeled with anti-PEDV polyclonal antibody (Green), respectively; the cell nuclei were stained with DAPI (blue). Scale bars, 50 µm.

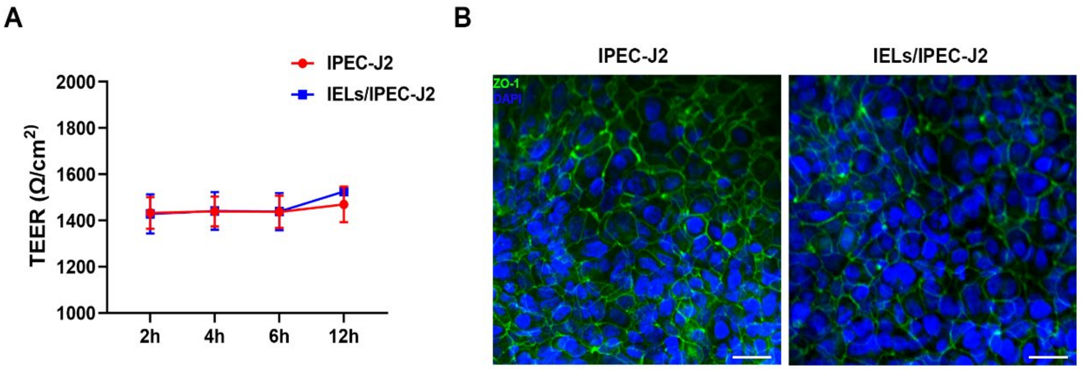

Supplementary Figure 3. Barrier integrity of IPEC-J2 monolayer after co-culturing with IELs. After IELs seeding, the barrier integrity of IPEC-J2 was evaluated. **A** TEER was measured using a voltohmmeter at different times after co-culturing. Data express mean ± SD from three samples. **B** After 12 h of co-culturing, the tight junction protein ZO-1 (green) was detected by Immunofluorescence. Bars: 20 µm.

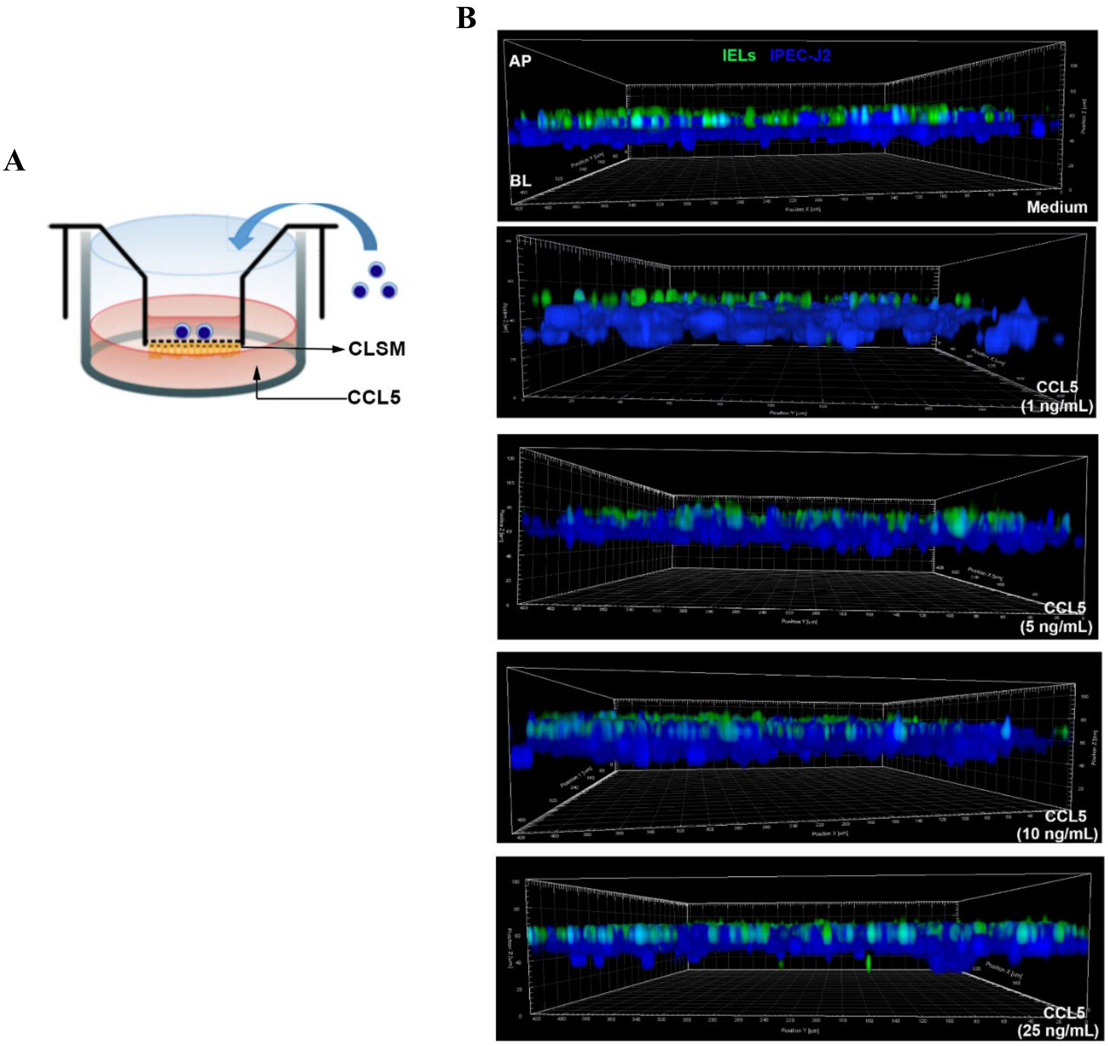

Supplementary Figure 4. CCL5 has no influence on transepithelial migration of intestinal intraepithelial lymphocytes (IELs). A Schematic of the experimental setting was used to study the migration of IELs influenced by chemokine CCL5. **B** A concentration gradient (1 ng/mL-25 ng/mL) of Chemokines CCL5 were added into the medium of the basolateral side. Filters from the co-culture system were determined via CLSM, and the intraepithelial and transepithelial (DAPI, blue) migration of IELs (CFSE, green) was shown by a three-dimensional (3D) rendering of representative fields. Bars, 10 μm.

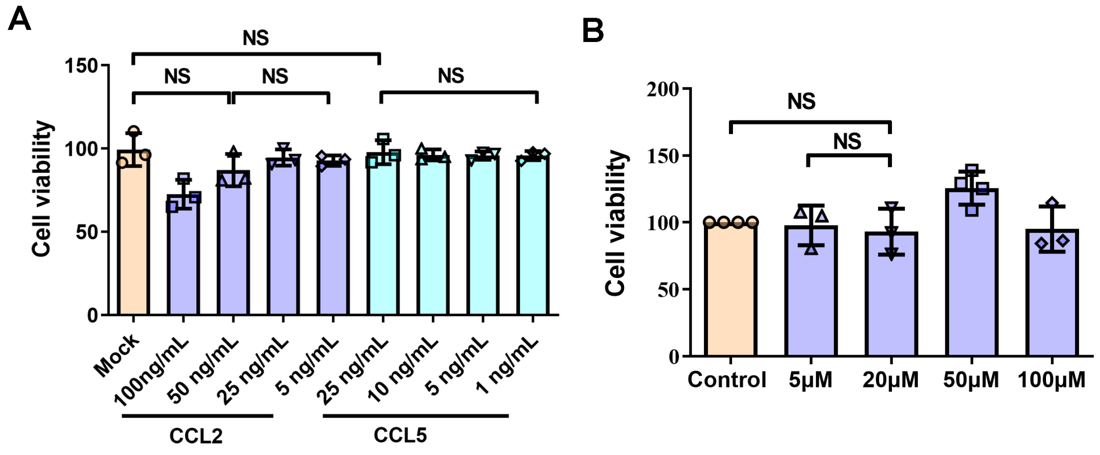

Supplementary Figure 5. Cell viability assay after chemokines CCL2 and CCL5, as well as TC1 treatment. **A** Cell viability was determined by CCK-8 assay after treatment of the IPEC-J2 cells with different concentrations of CCL2 and CCL5 for 24 h. **B** Cell viability was determined by CCK-8 assay after treatment of the IPEC-J2 cells with different concentrations of CCL2 inhibitor TC1.

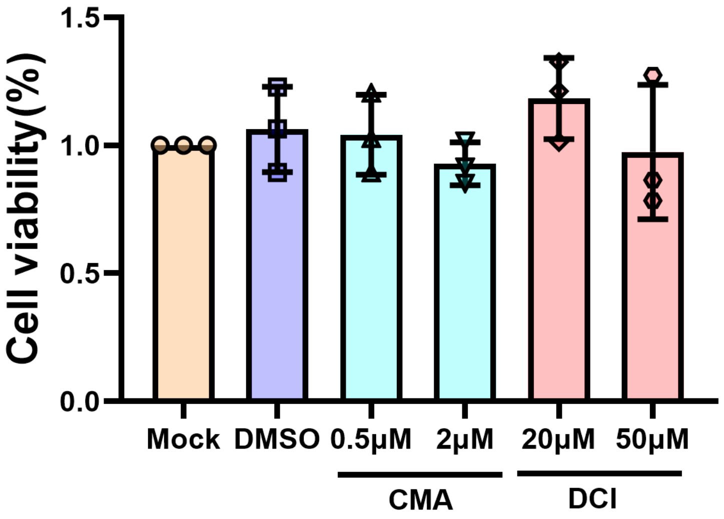

Supplementary Figure 6. Cell viability assay after CMA and DCI treatment. Cell viability was determined by CCK-8 assay after treatment of the IELs with different concentrations of inhibitors for perforin (CMA) and granzyme B (DCI).

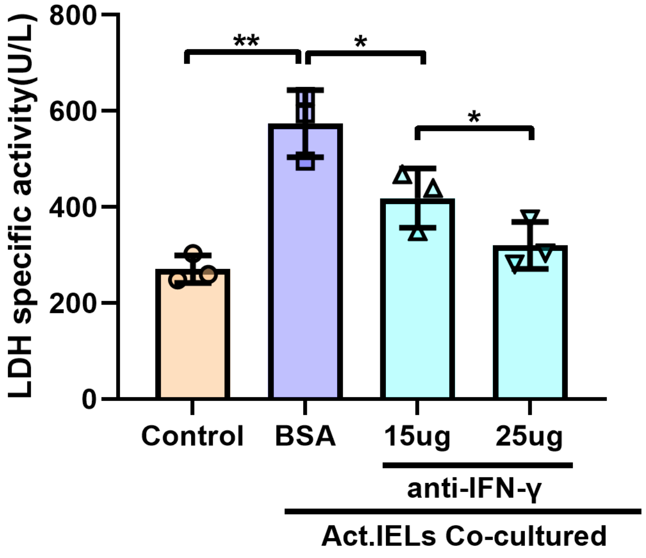

Supplementary Figure 7. The IELs-induced apoptosis of epithelial cells was significantly inhibited by neutralization of interferon-γ. Mock or whole inactivated virus (WIV) porcine epidemic diarrhea virus (PEDV) pre-activated IELs were co-cultured with PEDV-infected epithelial cells for 24 h. During co-cultivation, blocking antibodies against IFN-γ were added to the medium with a certain concentration gradient. The extracellular LDH activity in each experimental group were determined.

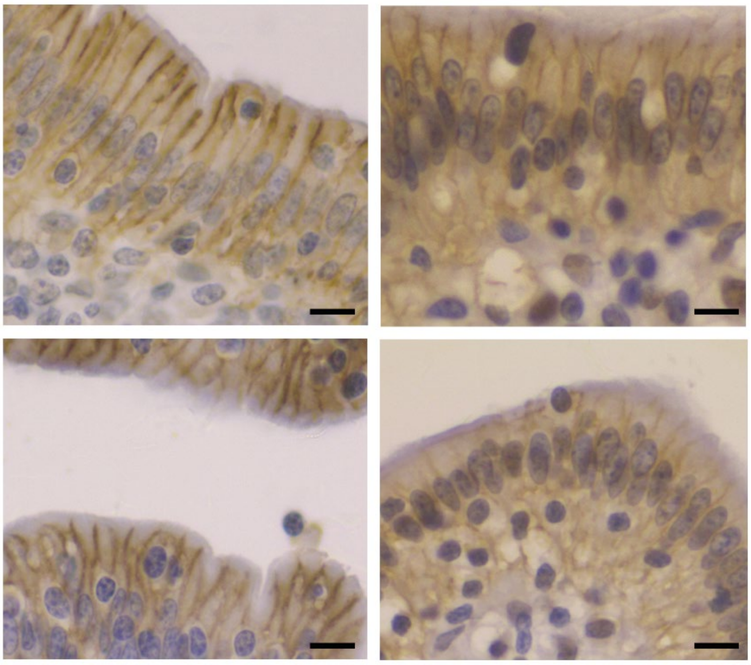

Supplementary Figure 8. Immunohistochemical analysis of intestinal tight junction. Immunohistochemistry staining showed that the intercellular movement of the IELs appeared to have no influence on the expression of the tight junctions in intestinal epithelium. The scale bar represents 10 μm.

5d

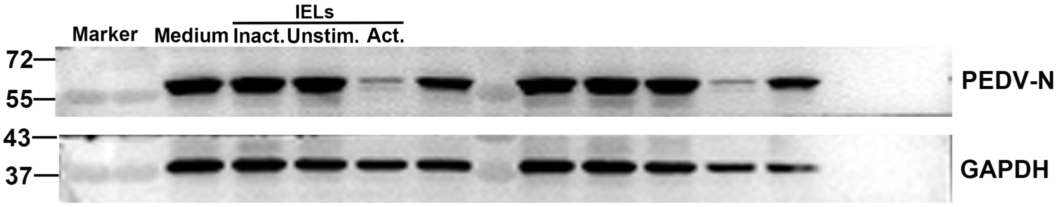

5g

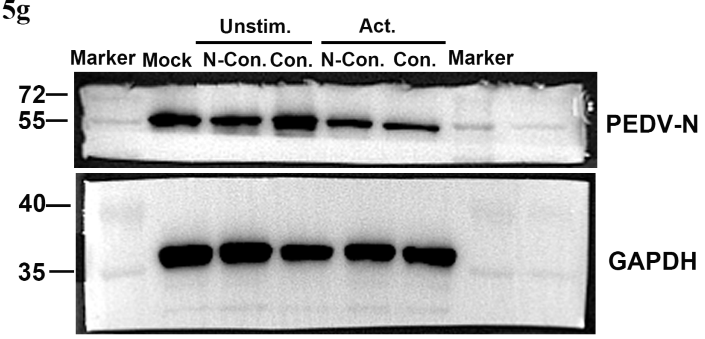

Supplementary Figure 9. Full western blots for Figures 5d and 5g.

Supplementary Table 1. Primers used for real-time PCR

Genes	Primers	Sequence (5'-3')*
PEDV (N)	Forward	CACCTCCTGCTTCACGTACA
	Reverse	AGCTCCACGACCCTGGTTAT
GAPDH	Forward	TCATCATCTCTGCCCCTTCT
(Sus scrofa)	Reverse	GTCATGAGTCCCTCCACGAT
GAPDH	Forward	ACATCATCCCTGCCTCTACTG
(Chlorocebus sabaeus)	Reverse	CCTGCTTCACCACCTTCTTG
CCL2	Forward	TGAATTCCCCAGTCACCTGC
(Chlorocebus sabaeus)	Reverse	TTGGGACACTTGGTGCTGTT
CCL5	Forward	ACGCTTAGCTGCTGTTTTGG
(Chlorocebus sabaeus)	Reverse	GCTCAGGCTGGCCCTTTTAT
IFN-γ	Forward	GGTGCGGATGCCAACTAATCT
(Sus scrofa)	Reverse	GTAAGTCTTCACCTGGGCGG
IFN-γ	Forward	TCGAATGTCCAACGCAAAGC
(Chlorocebus sabaeus)	Reverse	TTGGGATGCTCTTCGACCTC
Mx2	Forward	CTTTCAAACGCATCCATGTTTC
(Chlorocebus sabaeus)	Reverse	GGTGGCTCTCCCTTATTTGTTCT
IFIT3	Forward	GCTCTTGAGAAGGGACTGAATC
(Chlorocebus sabaeus)	Reverse	CAGCATCAGGGTCTTCCTTAC
OAS3	Forward	CTGGTCTGAGCCTCAAGTTTC
(Chlorocebus sabaeus)	Reverse	GGCTAACATCCATCCAGTCTTC
Viperin	Forward	CCTGTTTGGTGCCTGAATCTA
(Chlorocebus sabaeus)	Reverse	CTTCCGTCCCTTTCTACAGTTC
IFITM1	Forward	GTCCCTGTTCAACACCATCTT
(Chlorocebus sabaeus)	Reverse	TCACATCGCCAACCATCTTC
ISG15	Forward	GGCTTATAACAGGGCTGGCT
(Chlorocebus sabaeus)	Reverse	TCAGGTCCCAGCTCATGG
