# Supplementary material for: Porcine intraepithelial lymphocytes undergo migration and produce an antiviral response following intestinal virus infection
Source: Commun Biol. 2022 Mar 22;5:252. doi: 10.1038/s42003-022-03205-2 (PMC8941121; doi:10.1038/s42003-022-03205-2)
Supplement: Supplementary file 3 — Description of additional supplementary items [file 42003_2022_3205_MOESM3_ESM.pdf]

Description of Additional Supplementary Files

File name: Supplementary Data 1

Description: Source data behind the graphs in the paper
