# Supplementary material for: Porcine intraepithelial lymphocytes undergo migration and produce an antiviral response following intestinal virus infection
Source: Commun Biol. 2022 Mar 22;5:252. doi: 10.1038/s42003-022-03205-2 (PMC8941121; doi:10.1038/s42003-022-03205-2)
Supplement: Supplementary file 5 — Reporting Summary [file 42003_2022_3205_MOESM5_ESM.pdf]

nature portfolio

Corresponding author(s): Qian Yang

Last updated by author(s): Jan 25, 2022

Reporting Summary

Nature Portfolio wishes to improve the reproducibility of the work that we publish. This form provides structure for consistency and transparency in reporting. For further information on Nature Portfolio policies, see our Editorial Policies and the Editorial Policy Checklist.

Statistics

For all statistical analyses, confirm that the following items are present in the figure legend, table legend, main text, or Methods section.

n/a	Confirmed	
☐	☒	The exact sample size (*n*) for each experimental group/condition, given as a discrete number and unit of measurement
☐	☒	A statement on whether measurements were taken from distinct samples or whether the same sample was measured repeatedly
☐	☒	The statistical test(s) used AND whether they are one- or two-sided<br>*Only common tests should be described solely by name; describe more complex techniques in the Methods section.*
☐	☒	A description of all covariates tested
☐	☒	A description of any assumptions or corrections, such as tests of normality and adjustment for multiple comparisons
☐	☒	A full description of the statistical parameters including central tendency (e.g. means) or other basic estimates (e.g. regression coefficient) AND variation (e.g. standard deviation) or associated estimates of uncertainty (e.g. confidence intervals)
☐	☒	For null hypothesis testing, the test statistic (e.g. *F*, *t*, *r*) with confidence intervals, effect sizes, degrees of freedom and *P* value noted<br>*Give P values as exact values whenever suitable.*
☒	☐	For Bayesian analysis, information on the choice of priors and Markov chain Monte Carlo settings
☒	☐	For hierarchical and complex designs, identification of the appropriate level for tests and full reporting of outcomes
☒	☐	Estimates of effect sizes (e.g. Cohen's *d*, Pearson's *r*), indicating how they were calculated

Our web collection on statistics for biologists contains articles on many of the points above.

Software and code

Policy information about availability of computer code

Data collection	Results are expressed as means ± SD and analyzed with SPSS 17.0.
Data analysis	One-way ANOVA was employed to determine statistical differences among multiple groups, and t-test was employed to determine similarities between two groups. *P < 0.05, **P <0.01. Data were combined from at least three independent experiments unless otherwisestated.

For manuscripts utilizing custom algorithms or software that are central to the research but not yet described in published literature, software must be made available to editors and reviewers. We strongly encourage code deposition in a community repository (e.g. GitHub). See the Nature Portfolio guidelines for submitting code & software for further information.

Data

Policy information about availability of data

All manuscripts must include a data availability statement. This statement should provide the following information, where applicable:

- Accession codes, unique identifiers, or web links for publicly available datasets
- A description of any restrictions on data availability
- For clinical datasets or third party data, please ensure that the statement adheres to our policy

The data that support the findings of this study are available from the corresponding author upon request.

Field-specific reporting

Please select the one below that is the best fit for your research. If you are not sure, read the appropriate sections before making your selection.

☒ Life sciences ☐ Behavioural & social sciences ☐ Ecological, evolutionary & environmental sciences

For a reference copy of the document with all sections, see nature.com/documents/nr-reporting-summary-flat.pdf

Life sciences study design

All studies must disclose on these points even when the disclosure is negative.

Sample size	All data are the mean ± SD, n= 3 per group. All results are representative of three independent experiments
Data exclusions	No data were excluded from the analyses
Replication	No data were excluded from the analyses
Randomization	For the PEDV infection experiment, piglets (one-month-old) with similar weight were allocated to two groups (3 piglets per group) with a completely random design using the random number generation function (Excel, Microsoft Corporation)
Blinding	The investigators were blinded to group allocation during data collection and analysis

Reporting for specific materials, systems and methods

We require information from authors about some types of materials, experimental systems and methods used in many studies. Here, indicate whether each material, system or method listed is relevant to your study. If you are not sure if a list item applies to your research, read the appropriate section before selecting a response.

Materials & experimental systems

n/a	Involved in the study	
☐	☒	Antibodies
☐	☒	Eukaryotic cell lines
☒	☐	Palaeontology and archaeology
☐	☒	Animals and other organisms
☒	☐	Human research participants
☒	☐	Clinical data
☒	☐	Dual use research of concern

Methods

n/a	Involved in the study	
☒	☐	ChIP-seq
☐	☒	Flow cytometry
☒	☐	MRI-based neuroimaging

Antibodies

Antibodies used	anti-pig PerCP-Cy5.5-CD3ε (9108764), anti-Pig PE-TCR γδ (938914), anti-Pig PE-Cy7-CD4 (0057746), anti-Pig FITC-CD8α (9149562) were purchased from BD Biosciences (San Jose, CA, USA), anti-pig CD3 (ab16669) and anti-pig CCL2 mAb antibody were purchased from Abcam (Cambridge, MA, USA), anti-pig epithelial cell marker PE- Keratin 18 (CK18) mAb (1:200, NBP1-97715PE) were purchased from Novus Biologicals, anti-PEDV N protein mAb were purchased from Medgene labs (Brookings, SD, USA), anti-mouse tight junction protein zonula occludens protein (ZO-1) (Z01-1A12), and secondary antibodies used for IFA, such as goat anti-mouse Alexa Fluor 488 (cat. no. A-11029) and goat anti-rabbit Alexa Fluor 488 (A32731) were purchased from Invitrogen (Carlsbad, CA, USA)
Validation	Li Y, Wu Q, Huang L, et al. An alternative pathway of enteric PEDV dissemination from nasal cavity to intestinal mucosa in swine. Nature communications, 2018, 9(1): 1-14.<br>Huang, L. , Wang, J. , Wang, Y. , Zhang, E. , & Yang, Q. . (2019). Upregulation of CD4+CD8+ memory cells in the piglet intestine following oral administration of bacillus subtilis spores combined with pedv whole inactivated virus. Veterinary Microbiology,235.<br>The website for some used antibodies<br>https://www.bdbiosciences.com/zh-cn/search-results?searchKey=561486 (anti-Pig PE-TCR γδ)<br>https://www.bdbiosciences.com/zh-cn/search-results?searchKey=551303 (anti-Pig FITC-CD8α)<br>https://www.bdbiosciences.com/zh-cn/search-results?searchKey=561473 (anti-Pig PE-Cy7-CD4)<br>https://www.bdbiosciences.com/zh-cn/search-results?searchKey=561478 (anti-pig PerCP-Cy5.5-CD3ε)<br>https://www.novusbio.com/products/cytokeratin-18-antibody-rge53_nbp1-97715pe [illegible]anti-pig PE- Keratin 18 (CK18) mAb[illegible]

Eukaryotic cell lines

Policy information about cell lines

Cell line source(s)	Marc-145 cells ,Vero E6 cell and IPEC-J2 cell lines were preserved in our laboratory

Authentication	Monkey and porcine cell lines are not applicable for STR validated<br>These two cells has been used for a series of related studies in our or other laboratory, including PEDV isolation, propagation and pathogenesis research.<br>Li Y, Wu Q, Huang L, et al. An alternative pathway of enteric PEDV dissemination from nasal cavity to intestinal mucosa in swine[J]. Nature communications, 2018, 9(1): 1-14.<br>Zhang S, Cao Y, Yang Q. Transferrin receptor 1 levels at the cell surface influence the susceptibility of newborn piglets to PEDV infection[J]. PLoS Pathogens, 2020, 16(7): e1008682.<br>Li Y, Wang G, Wang J, et al. Cell attenuated porcine epidemic diarrhea virus strain Zhejiang08 provides effective immune protection attributed to dendritic cell stimulation[J]. Vaccine, 2017, 35(50): 7033-7041.<br>Sun M, Yu Z, Ma J, et al. Down-regulating heat shock protein 27 is involved in porcine epidemic diarrhea virus escaping from host antiviral mechanism[J]. Veterinary microbiology, 2017, 205: 6-13.
Mycoplasma contamination	Confirmed no mycoplasma contamination
Commonly misidentified lines<br>(See ICLAC register)	NA

Animals and other organisms

Policy information about studies involving animals; ARRIVE guidelines recommended for reporting animal research

Laboratory animals	The newborn (0 day), one-month-old, and six-month-old Duroc × Landrace × Yorkshire female piglets were obtained from a swine herd at the Jiangsu Academy of Agricultural Science and were raised in highly sanitary conditions in the experimental animal center of Nanjing Agricultural University. The swine herd was seronegative for antibodies against PEDV, porcine reproductive and respiratory syndrome virus (PRRSV), porcine respiratory corona virus (PRCV), transmissible gastroenteritis virus (TGEV), and porcine circovirus type 2. Each experimental pig group was housed in a separate room in a high-security isolation facility.
Wild animals	NA
Field-collected samples	NA
Ethics oversight	All animal procedures and experiments were performed according to protocols approved by the Institutional Animal Care and Use Committee of Nanjing Agricultural University (Nanjing, China) and followed the National Institutes of Health guidelines.

Note that full information on the approval of the study protocol must also be provided in the manuscript.

Flow Cytometry

Plots

Confirm that:

☒ The axis labels state the marker and fluorochrome used (e.g. CD4-FITC).

☒ The axis scales are clearly visible. Include numbers along axes only for bottom left plot of group (a 'group' is an analysis of identical markers).

☒ All plots are contour plots with outliers or pseudocolor plots.

☒ A numerical value for number of cells or percentage (with statistics) is provided.

Methodology

Sample preparation	IELs were isolated from porcine ileum tissue. Briefly, the small intestines were opened, freed of Peyer's patches, and washed in PBS. Mucus dissociation was performed by incubating tissues in 30 mL of Hank's balanced salt solution (HBSS) containing 5 mM dithiothreitol (Invitrogen 15508) and 2% heat inactivated fetal calf serum (FCS; Gibco A38401) for 20 min. The epithelial layer was gently scraped off using a scalpel, and incubated for 40 min in RPMI 1640 containing 10% FCS and 1 mM dithiothreitol in a turning wheel. The cells in the epithelial layer were collected by transferring tissue into 30 mL of HBSS containing 5 mM EDTA and 2% FCS. A total of 3 sequential incubations in fresh epithelial removal solution were performed for 25 min each, transferring the tissue to fresh solution for each incubation. Liberated cells from the epithelial removal and wash solutions were retained, pooled, passed through a 100-micron nylon filter, and washed with HBSS containing 2 mM L-glutamine and 2% FCS. Isolated cells were centrifuged in a 20%/40%/80% Percoll density gradient at 700 g for 30 min. The IELs were harvested from the 40% to 80% Percoll interface. After isolation, the cells were transferred to fresh plates and cultured with IL-2 (10 U mL-1) in RPMI 1640 Supplemented with 10% FBS, 100 U mL-1 penicillin, 100 µg mL-1 streptomycin, 2 mM glutamine, 1 mM sodium pyruvate, 2.5 mM HEPES, and non-essential amino acids (Invitrogen).
Instrument	BD FACSVerse
Software	FlowJo 10
Cell population abundance	The purity of the isolated IELs was identified via FACS analysis, in which more than 70% of cells expressed a CD3 receptor and were used for subsequentexperiments
Gating strategy	CD3ε was used to gate T cells group in IELs, and further characterization of total T cells by expression of cell surface markers

Gating strategy

γδTCR. Subsequently the expression of CD4, and CD8α was assessed in γδTCR+ or γδTCR- group. The boundary between positive and negative cells was determined by fluorescence-minus one (FMO) and isotype controls.

☒ Tick this box to confirm that a figure exemplifying the gating strategy is provided in the Supplementary Information.
